# Supplementary figures and images for: The Driverless Triple-Wild-Type (BRAF, RAS, KIT) Cutaneous Melanoma: Whole Genome Sequencing Discoveries
Source: Cancers (Basel). 2023 Mar 10;15(6):1712. doi: 10.3390/cancers15061712 (PMC10046270; doi:10.3390/cancers15061712)

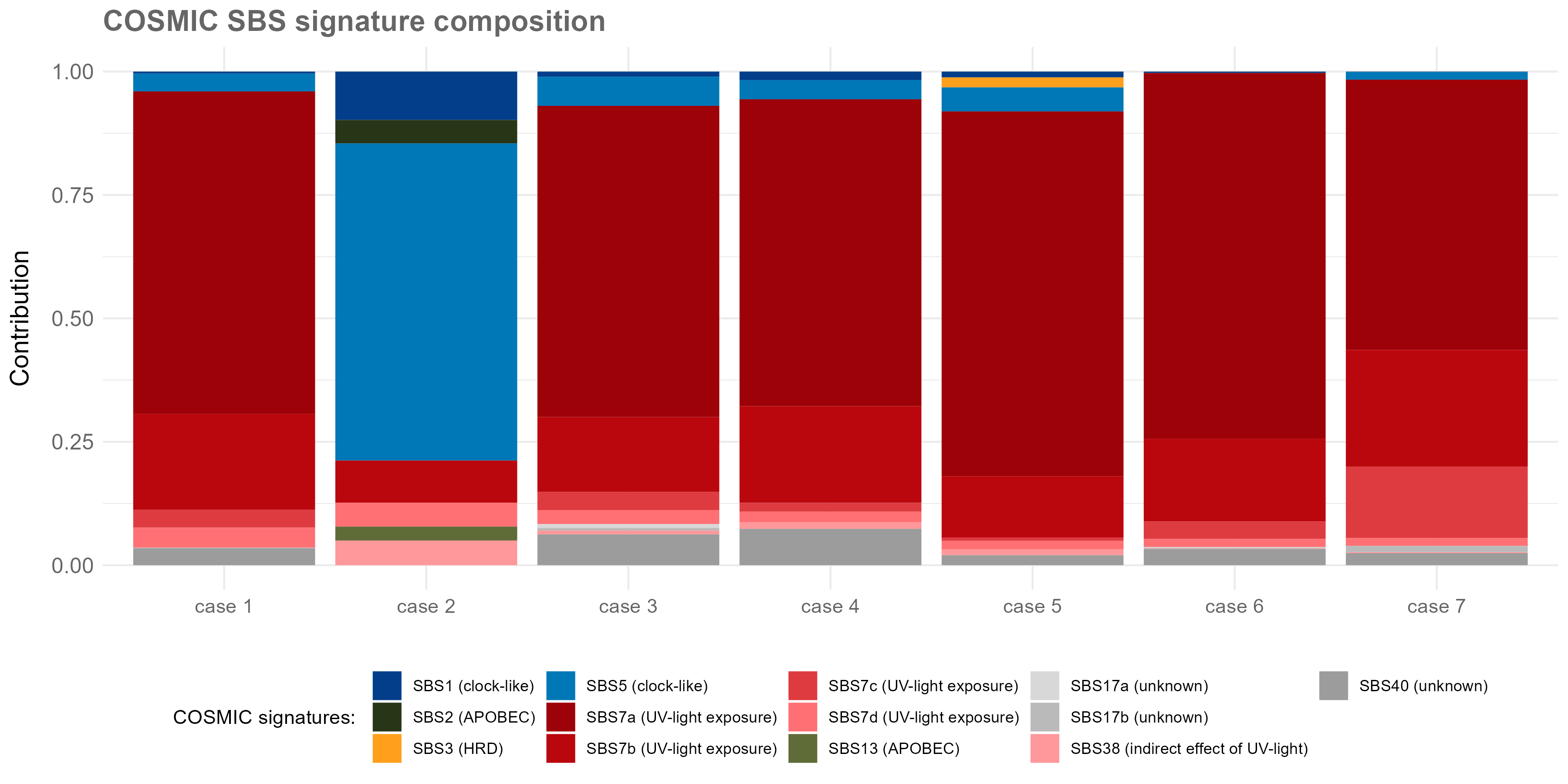

Supplement: Supplementary file 1 [file cancers-15-01712-s001.zip › cancers-2218166-supplementary/cancers-2218166-Figure S1.png]

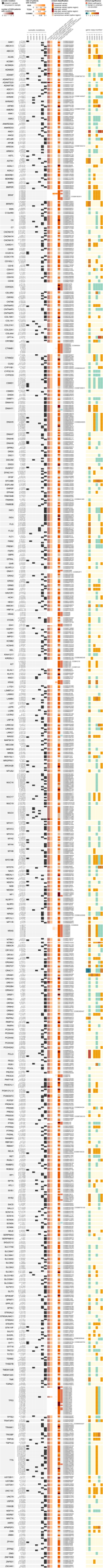

Supplement: Supplementary file 1 [file cancers-15-01712-s001.zip › cancers-2218166-supplementary/cancers-2218166-Table S1.pdf]
